# Interleukin-31 Signaling Bridges the Gap Between Immune Cells, the Nervous System and Epithelial Tissues

**DOI:** 10.3389/fmed.2021.639097

**Published:** 2021-02-10

**Authors:** Jana Maria Nemmer, Marcus Kuchner, Angeliki Datsi, Péter Oláh, Valérie Julia, Ulrike Raap, Bernhard Homey

**Affiliations:** ^1^Department of Dermatology, Medical Faculty, University Hospital Düsseldorf, Heinrich-Heine-University Düsseldorf, Düsseldorf, Germany; ^2^Medical Faculty, Institute for Transplantation Diagnostics and Cell Therapy, University Hospital Düsseldorf, Heinrich-Heine-University Düsseldorf, Düsseldorf, Germany; ^3^Department of Dermatology, Venereology and Oncodermatology, Medical Faculty, University of Pécs, Pécs, Hungary; ^4^Galderma Pharmaceuticals, Vevey, Switzerland; ^5^Division of Experimental Allergy and Immunodermatology, Department of Dermatology, University of Oldenburg, Oldenburg, Germany

**Keywords:** atopic dematitis, interleukin-31, interleukin-31 receptor, neuroinflammation, pruritus

## Abstract

Pruritus represents one of the most common symptoms in dermatology and general medicine. Chronic pruritus severely impairs the quality of life of affected patients. During the last two decades a number of modulators and mediator of pruritus have been identified. Recently, Interleukin (IL)-31 and its receptor complex attracted significant interest, as clinical phase two studies demonstrated therapeutic efficacy of the neutralizing IL-31 receptor A (IL-31RA) antibody nemolizumab in patients suffering from atopic dermatitis or prurigo nodularis. IL-31 has also been shown to play relevant roles in allergic contact dermatitis, urticaria, mastocytosis, allergic rhinitis and asthma. Here, we summarize the current knowledge of the novel cytokine IL-31 and its receptor regarding cellular origin, regulation, signaling pathways and their involvement in biological processes such as pruritus, neuronal growth, inflammation, barrier dysfunction and tissue remodeling.

## Introduction

Pruritus represents an important archaic sensation, and its evolutionary role is to sensitize the host to a distinct body site in order to remove invading parasites or plant matter. Chronic pruritus severely impairs the quality of life of affected patients and represents a significant unmet medical need. In 2004, Dillon et al. first demonstrated the involvement of IL-31 signaling in the development of pruritus and atopic dermatitis-like skin lesions (1). Subsequently an emerging body of evidence supported a central role of IL-31 and its receptor in bridging the immune system with neurons, epithelial surfaces and connective tissue. Recently, phase two clinical trials demonstrated therapeutic efficacy of the neutralizing IL-31RA antibody nemolizumab in patients suffering from atopic dermatitis or prurigo nodularis (2, 3). In addition, IL-31 also plays a role in T_H_2-driven and autoimmune diseases such as contact dermatitis, urticaria, mastocytosis, allergic rhinitis; but also systemic sclerosis, dermatomyositis, and lupus erythematosus (4–12). Here, we summarize the current knowledge on the novel cytokine IL-31 and its receptor regarding cellular origin, regulation, signaling pathways and their involvement in biological processes such as pruritus, neuronal growth, inflammation, barrier dysfunction and tissue remodeling.

## Interleukin-31

IL-31 represents a member of the IL-6 family of cytokines, which share a four-helical structure and the majority signals through receptor complexes containing glycoprotein 130 (gp130). This family consists of nine members including IL-6, IL-11, ciliary neurotrophic factor (CNTF), leukemia inhibitory factor (LIF), oncostatin M (OSM), cardiotrophin 1 (CT-1), cardiotrophin-like cytokine (CLC), IL-27 and IL-31. Members of the IL-6 family are predominantly expressed under proinflammatory conditions and realize pleiotropic functions in immune-related processes (13). The *IL31* gene is located on chromosome 12q24.31 (1). Recent studies support the production of IL-31 in a variety of leukocyte subsets including T cells, eosinophils, basophils, mast cells, monocytes and dendritic cells. However, it is widely accepted that effector memory T cells with a T_H_2 phenotype represent the major source of IL-31 (4, 14–18). Interestingly, findings linking IL-31 expression with patients suffering from *Dedicator of cytokinesis protein 8* (*DOCK8*) deficiency-related hyper IgE syndrome pointed to an important upstream regulation pathway. *DOCK8* loss-of-function mutations lead to a combined immunodeficiency with elevated serum levels of IgE, eosinophilia, decreased number of B and T cells as well as severe atopic dermatitis with increased IL-31 expression (19). Subsequent *in vitro-* as well as *in vivo*-studies demonstrated that DOCK8 is a negative regulator of the nuclear translocation of Endothelial PAS domain-containing protein 1 (EPAS1). This function is dependent of STK4 (MST1), a serine threonine kinase involved in apoptosis (20, 21). Knockdown of the *MST1* gene led to an increased translocation of EPAS1 to the nucleus in alignment with *DOCK8* knockout models showing elevated IL-31 expression (21). Consequently, clinical symptoms of patients with STK4 (*MST1*) mutations leading to deficiency at the protein level are resembling those with DOCK8 deficiency (22). EPAS1 is regulated by IL-4-mediated signal transducer and activator of transcription 6 (STAT6) signaling in CD4^+^ T cells (23). Jabara et al. reported that DOCK8 is constitutively associated with myeloid differentiation primary response protein (MyD88), an adaptor protein of Toll-like receptors (TLR) (24). Hence it is interesting to speculate whether microbes such as *S. aureus* may influence IL-31 production through TLR engagement.

### Interleukin-31 Receptor

Within the IL-6 family IL-31 is special, since it shares the four helical structure but does not signal through a receptor complex containing gp130. Instead, it binds to a heterodimeric receptor composed of the IL-31RA chain and the oncostatin M receptor (OSMR) β chain. The *IL31RA* gene is located on chromosome 5q11.2, 24 kb downstream of *IL6ST* (25, 26). From a phylogenetical view, IL-31RA is paralogous to gp130, although they share only 28% amino acid identity. It has five fibronectin type III (FNIII)-like domains and shares the WSxWS motif and the conserved cysteines with other type I cytokine receptors within the cytokine binding domain [as reviewed in (27)]. Horejs-Hoeck et al. showed that STAT1 is a relevant transcription factor to activate the promoter region of the *IL31RA* gene following IFN-γ stimulation and this regulation pathway was confirmed in several studies and cell types (28). Cytokine effects are based on their capacity to assemble receptor complexes to bring the associated kinases in spatial proximity for phosphorylation. Therefore, the expression pattern of relevant receptor chains in target cells determines their ability to respond to specific cytokine signals. The OSMRβ chain is considered to be widely expressed (29). Hence the limiting factor for IL-31 signal transduction appears to be the expression of the IL-31RA chain. Recent studies demonstrate that multiple leukocyte subsets, as well as epithelial and stromal cells express IL-31RA in steady state or more importantly under activated conditions (14, 17, 28, 30, 31). At first, the expression of IL-31RA on itch-conducting dorsal root ganglia (DRG) neurons attracted significant attention (4). Non-immune cells such as keratinocytes, fibroblasts and a distinct subset of DRG neurons also express and signal via IL-31RA (18, 31, 32). Binding of IL-31 to the receptor complex leads to phosphorylation of STAT1, STAT3 and STAT5 via the associated Janus kinase (JAK) 1 and JAK2 (33, 34). Besides JAK/STAT signaling the IL-31 receptor complex activates MEK/ERK and PI3K/Akt pathways as well as the JNK pathway (33, 35–37). Negative feedback mechanisms of IL-31RA signaling include suppressor of cytokine signaling (SOCS)1- and SOCS3-dependent inhibition of STAT3 activation (34). Interestingly, OSMR is a shared subunit of the receptor complexes of IL-31 and OSM, although their biological functions differ. While IL-31 is involved in many T_H_2-driven diseases as mentioned above, OSM plays an important role in hematopoiesis and cancer development (38). It will be of interest to elucidate the distinct roles of IL-31 and OSM. Taken together, the diverse distribution of its receptor enables IL-31 to target the nervous system, immune functions, epithelial surfaces and stromal cells.

### Nervous System

**Figure 1:**
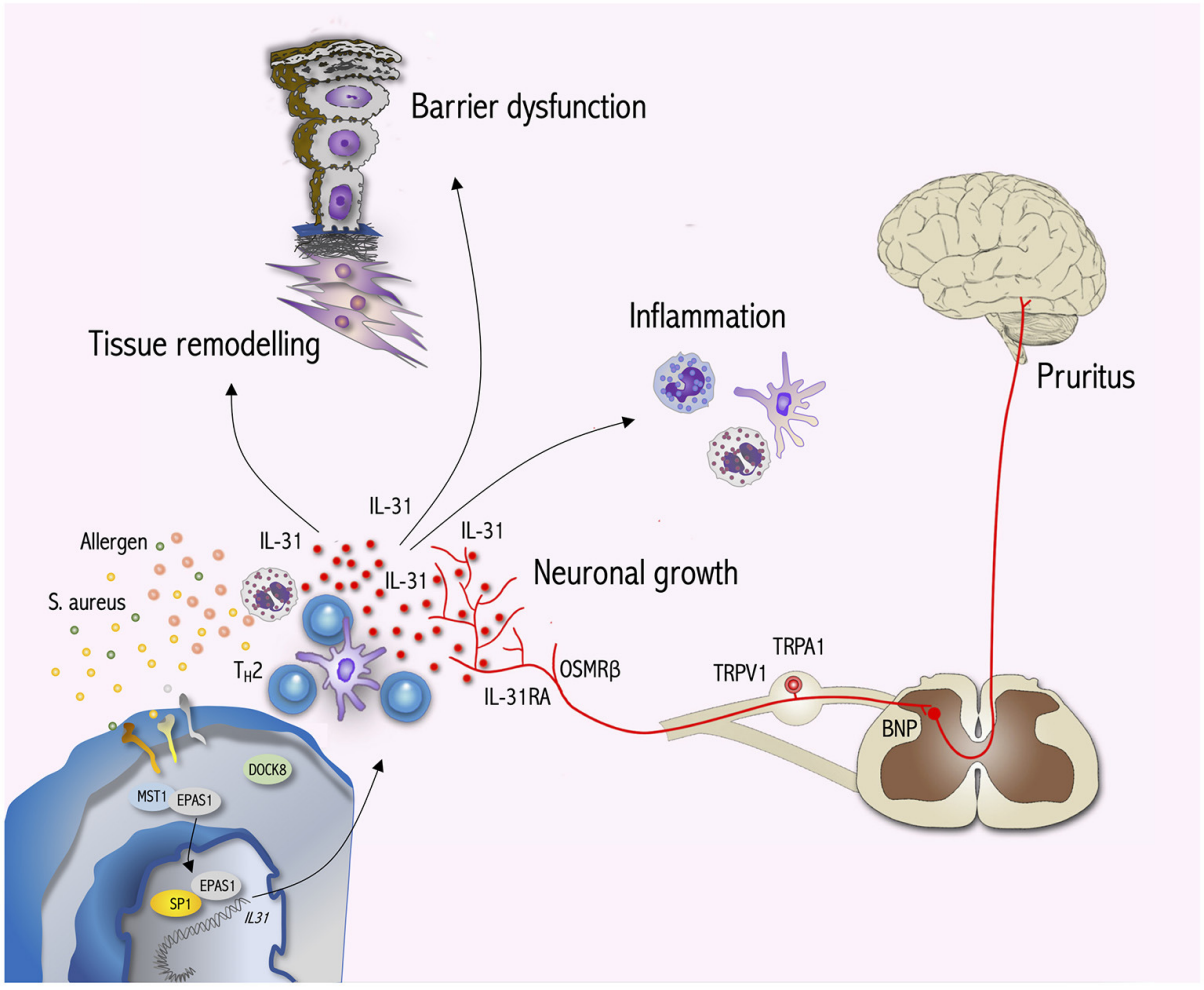
IL-31 signaling bridges the gap between the immune system, neurons and epithelial surfaces. During T cell activation DOCK8 dissociates from EPAS1 enabling EPAS1 to translocate to the nucleus. Within the nucleus EPAS1 forms a complex with SP1 initiating *IL31* transcription. T_H_2 cells are a major source of IL-31 production. In IL-31RA/OSMRß-expressing sensory neurons IL-31 induces the activation of ion channels (TRPV1, TRPA1) and transmits pruritus signals via BNP to the CNS. Moreover, IL-31 stimulates neuronal growth and the branching of sensory nerves. Furthermore, IL-31 targets immune cells such as mast cells, eosinophils, basophils and monocytes/dendritic cells to induce inflammation. Within the skin, IL-31 impairs keratinocyte differentiation as well as barrier function and in turn activates keratinocytes to produce cytokines, chemokines and pruritus mediators amplifying skin inflammation and itch. Interestingly, IL-31 also interacts with dermal fibroblasts initiating tissue remodeling by inducing collagen production and cytokine as well as chemokine expression. Hence, IL-31 signaling exerts pleiotropic effects beyond pruritus.

Within the cytokine superfamily IL-31 has a unique position, because it bridges the gap between the immune and the peripheral nervous systems (see Figure 1). During recent years, several independent studies confirmed the expression and signaling of IL-31RA and OSMRβ in a subset of murine as well as human DRG neurons (4, 18, 39–41). These findings stimulated further research on IL-31 targeting sensory neurons. Cevikbas et al. demonstrated in murine behavioral studies that IL-31 induces itch but not pain and mediates its effects independent of mast cells by activating the ion channels TRPV1 and TRPA1. In DRG neurons IL-31 induces intracellular Ca^2+^ mobilization as well as STAT3 and ERK phosphorylation (18). Following the activation of afferent DRG neurons, neurotransmitters such as natriuretic polypeptide b (Nppb) forward the signal further to the dorsal horn of the spinal cord, where the gastrin-releasing peptide receptor (Grpr) system is subsequently activated transmitting the signal further to projection neurons that transport the information to the brain (42–45). Recently, Meng et al. showed that IL-31 stimulation increased *Nppb* in DRG neurons *in vitro* and *in vivo* and induced soluble *N*-ethylmaleimide-sensitive-factor attachment receptor (SNARE)–dependent brain natriuretic peptide (BNP) release (46). In pharmacological studies, Ma et al. demonstrated that activation of the spinal neuropeptide Y system dampens IL-31-induced scratching behavior through activation of neuropeptide Y_2_ receptor on DRG neurons (47). Notably, noxious signals activate neuropeptide Y interneurons, and this may explain, how the infliction of pain, e.g., through scratching, heat, cold, etc., may reduce itch perception in atopic dermatitis patients. Next to the initiation of pruritus signals, Feld et al. recently demonstrated that IL-31 also induces a distinct transcriptional program in sensory neurons, leading to nerve elongation and branching both *in vitro* and *in vivo*. Hence the increased density of neuronal networks in the skin may help us understand why atopic dermatitis patients experience increased sensitivity to minimal stimuli inducing sustained itch (48).

### Immune Functions

Since the IL-31 receptor heterodimer is expressed on a variety of different leukocyte subsets including monocytes, macrophages, dendritic cells, eosinophils, mast cells and basophils, it is interesting to have a closer look at immune functions that are targeted by IL-31. Recently, Raap et al. demonstrated that basophils upon IL-31 stimulation do not release histamine but secrete large amounts of IL-4 and IL-13 (14). This is of particular importance since IL-4 is a critical factor for the differentiation of T cells into a T_H_2 phenotype and the source of IL-4 in this dendritic cell-driven process is still debated (49, 50). Thus, IL-31 secretion may serve as an early upstream signal during the development of type 2 skin inflammation. In eosinophils and dendritic cells IL-31 induced a set of proinflammatory cytokines and chemokines including TNF-α, IL-1β, IL-6, CXCL1, CXCL8, CCL2 and CCL5 and CCL22 (16, 51). Through these molecules, IL-31 may recruit neutrophils (CXCL1, CXCL8), dendritic cells (CCL2), T_H_1 (CCL5) and T_H_2 (CCL22) cells to sites of inflammation or promote angiogenesis (CXCL1, CXCL8) and tissue remodeling (CCL2, IL-6) (52, 53). On the other hand, cytokines such as TNF-α, IL-1β, and IL-6 may directly affect T cell, B cell and dendritic cell functions as well as activate surrounding stromal or epithelial cells (54). Hence, IL-31 signaling is able to amplify inflammation via different self-reinforcing loops.

### Epithelial Surfaces

In 2004, Dillon et al. demonstrated for the first time the expression of IL-31RA and OSMRß in epidermal keratinocytes and IL-31 stimulation resulted in chemokine (CXCL1, CCL1, CCL4, CCL17, CCL19, CCL22, and CCL23) expression (1). Subsequently, a number of studies confirmed IL-31 receptor expression on keratinocytes and showed downstream signaling leading to STAT3 and ERK phosphorylation. Recent findings in 2D and 3D keratinocyte culture systems unravel, that IL-31 stimulation also modulates keratinocyte differentiation and disrupts epithelial barrier function (55–57). Cornelissen et al. demonstrated that IL-31 induced cell cycle arrest in keratinocytes and inhibited proliferation (55). Moreover, IL-31 elicited a differentiation defect with decreased filaggrin expression and impaired barrier functions facilitating transepidermal allergen penetration in organotypic keratinocyte cultures (55–57). Next to the production of chemokines, IL-31-stimulated keratinocytes contribute to skin inflammation through the expression of key proinflammatory mediators including IL-1α, IL-1β, IL-6, S100A7, S100A8, S100A9, β-defensin-2, β-defensin-3 (57). Moreover, IL-31-induced BNP release from sensory neurons may activate keratinocytes to produce proinflammatory cytokines and chemokines (46). Hence, inflammation circuits between epithelial surfaces, nerves and immune cells are connected and amplified via IL-31 signaling.

More recently, another keratinocyte-driven circuit potentially amplifying pruritus has been proposed. Andoh et al. demonstrated that intradermal injection of IL-31 induced thromboxane synthase in epidermal keratinocytes and significantly increased the concentration of thromboxane B_2_, a metabolite of the pruritus mediator thromboxane A_2_ (58). Moreover, keratinocytes produced the pruritus mediator leukotriene B_4_ (LTB4) following IL-31 treatment and the LTB4 receptor antagonist CMHVA as well as the 5-lipoxygenase inhibitor, zileuton, suppressed the scratching behavior of mice intradermally injected with IL-31 (59).

Thus, next to the direct engagement of peripheral sensory neurons, IL-31 may sustain pruritus via keratinocyte activation and the release of other pruritus mediators (58–60). Notably, besides epidermal keratinocytes, bronchial and gut epithelial cells have been shown to be a target of IL-31 (61, 62).

### Tissue Remodeling

Given the pleiotropic functions of IL-6 family members it has been reasonable to also investigate the role of IL-31 in tissue remodeling. Several studies report a direct effect of IL-31 on fibroblasts (17, 63). IL-31 signaling resulted in STAT3 phosphorylation and the activation of ERK, JNK and AKT (17). It is important to note that pro-fibrotic processes often follow STAT3 signaling pathways representing a considerable checkpoint for tissue fibrosis (64). Indeed, high levels of IL-31 were reported in plasma, fibrotic skin and lung lesions of systemic sclerosis (SSc) patients (10). Moreover, IL-31RA was upregulated in fibrotic skin and lung fibroblasts. Gene expression analysis of IL-31-treated dermal fibroblasts revealed a total of 561 differentially expressed genes with 200 genes involved in processes such as cell proliferation and growth. Furthermore, the authors showed that IL-31 stimulated dermal fibroblast activated STAT3 and PI3K/Akt pathways and induced collagen I production (10). Several expression studies in fibroblasts also support that IL-31 stimulation promotes inflammation and tissue remodeling through the induction of IL-6, IL-16, IL-32, CCL2, CCL13, CCL15, CXCL1, CXCL3, CXCL8, and CXCL10 and matrix metalloproteinases (MMP-1, MMP-3, MMP-7 and MMP-25) (63).

Taken together, IL-31 represents a master regulator of neuroimmune inflammation and bridges the gap between immune cells, the nervous system and epithelial tissues.

## Discussion

**Figure 2:**
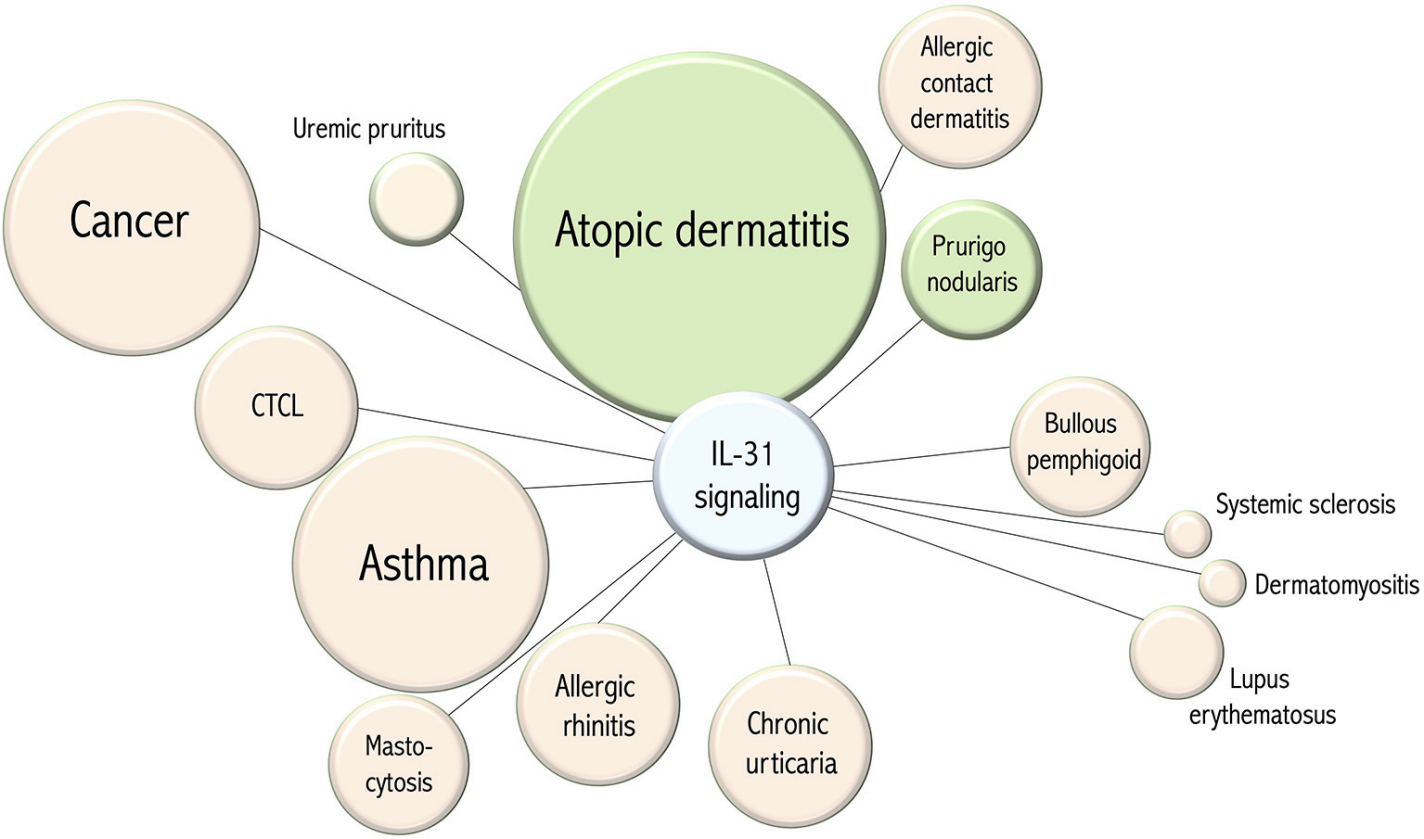
Disease associations of IL-31 signaling. The circle diameter of each item correlates to the number of disease-associated publications listed in PubMed (pubmed.ncbi.nlm.nih.gov). The green color of an item corresponds to the therapeutic efficacy in clinical trials of targeting IL-31 signaling. The distance of an item from the center indicates whether IL-31 signaling hypothetically could serve as a therapeutic target.

During recent years a variety of diseases have been associated with IL-31 signaling (see Figure 2). An initial focus was directed on processes accompanied with pruritus and following, at least partly, concepts of type 2 inflammation. These included atopic dermatitis, allergic contact dermatitis, urticaria, mastocytosis, allergic rhinitis and asthma (4–9). Given the role of IL-31 signaling in the development of itch it is interesting to speculate whether IL-31 may also be involved in the stimulation of sneezing, coughing or bronchial hyperreactivity. In this context, other epithelial surfaces such as the gut and conditions such as irritable bowel syndrome also come to mind. STAT1 related regulation of IL-31RA may link this pathway also with autoimmune diseases such as systemic sclerosis, dermatomyositis and lupus erythematosus (10–12). A subset of affected patients experience severe pruritus but IL-31 signaling in autoimmune inflammation may also facilitate fibrosis and amplify inflammatory circuits. These are interesting aspects that need to be further explored in the future. Among autoimmune skin diseases, bullous pemphigoid has a unique position since patients develop autoantibodies against hemidesmosomes (BP180, BP230), eosinophilia, urticarial skin lesions, blisters and suffer from severe pruritus. A number of studies demonstrated the expression of IL-31 and its receptor in this condition and bullous pemphigoid appears to be a very interesting candidate for clinical studies targeting IL-31 signaling (65–68). Early on IL-31 expression and serum levels have been investigated in patients suffering from cutaneous T cell lymphoma (69–71). Notably, an increasing body of literature links IL-31 with malignant diseases such as endometrial carcinoma, lung cancer, myeloproliferative disorders, mastocytosis, cutaneous T cell lymphoma and follicular B cell lymphoma (7, 69, 71–76). The role of IL-31 in malignant diseases remains largely obscure but this aspect is worth to closely follow in the future. Taken together, IL-31 is a neuroimmune cytokine and IL-31RA signaling represents a master regulator of inflammation that bridges the gap between immune cells, the nervous system and epithelial tissues.

## Author Contributions

JN, MK, AD, BH, and VJ conceptualization and writing. UR and BH critical revision of manuscript. BH and PO editing. All authors contributed to the article and approved the submitted version.

## Conflict of Interest

BH has received research funding from Galderma Pharmaceuticals and participates in studies with nemolizumab. VJ is an employee of Galderma Pharmaceuticals and is involved in nemolizumab development. The remaining authors declare that the research was conducted in the absence of any commercial or financial relationships that could be construed as a potential conflict of interest.
